# Genome-Wide Association Analysis of Stable Stripe Rust Resistance Loci in a Chinese Wheat Landrace Panel Using the 660K SNP Array

**DOI:** 10.3389/fpls.2021.783830

**Published:** 2021-12-22

**Authors:** Fangjie Yao, Fangnian Guan, Luyao Duan, Li Long, Hao Tang, Yunfeng Jiang, Hao Li, Qiantao Jiang, Jirui Wang, Pengfei Qi, Houyang Kang, Wei Li, Jian Ma, Zhien Pu, Mei Deng, Yuming Wei, Youliang Zheng, Xianming Chen, Guoyue Chen

**Affiliations:** ^1^Triticeae Research Institute, Sichuan Agricultural University, Chengdu, China; ^2^State Key Laboratory of Crop Gene Exploitation and Utilization in Southwest China, Sichuan Agricultural University, Chengdu, China; ^3^College of Agronomy, Sichuan Agricultural University, Chengdu, China; ^4^Wheat Health, Genetics and Quality Research Unit, United States Department of Agriculture, Agricultural Research Service, Pullman, WA, United States; ^5^Department of Plant Pathology, Washington State University, Pullman, WA, United States

**Keywords:** wheat landraces, resistance, stripe rust, GWAS, KASP markers

## Abstract

Stripe rust (caused by *Puccinia striiformis* f. sp. *tritici*) is one of the most severe diseases affecting wheat production. The disease is best controlled by developing and growing resistant cultivars. Chinese wheat (*Triticum aestivum*) landraces have excellent resistance to stripe rust. The objectives of this study were to identify wheat landraces with stable resistance and map quantitative trait loci (QTL) for resistance to stripe rust from 271 Chinese wheat landraces using a genome-wide association study (GWAS) approach. The landraces were phenotyped for stripe rust responses at the seedling stage with two predominant Chinese races of *P. striiformis* f. sp. *tritici* in a greenhouse and the adult-plant stage in four field environments and genotyped using the 660K wheat single-nucleotide polymorphism (SNP) array. Thirteen landraces with stable resistance were identified, and 17 QTL, including eight associated to all-stage resistance and nine to adult-plant resistance, were mapped on chromosomes 1A, 1B, 2A, 2D, 3A, 3B, 5A, 5B, 6D, and 7A. These QTL explained 6.06–16.46% of the phenotypic variation. Five of the QTL, *QYrCL.sicau-3AL*, *QYrCL.sicau-3B.4*, *QYrCL.sicau-3B.5*, *QYrCL.sicau-5AL.1* and *QYrCL.sicau-7AL*, were likely new. Five Kompetitive allele specific PCR (KASP) markers for four of the QTL were converted from the significant SNP markers. The identified wheat landraces with stable resistance to stripe rust, significant QTL, and KASP markers should be useful for breeding wheat cultivars with durable resistance to stripe rust.

## Introduction

Stripe rust (also called yellow rust), caused by *Puccinia striiformis* f. sp. *tritici* (*Pst*), is a serious disease of wheat worldwide. The fungal pathogen produces yellow to orange-colored uredinia mainly on leaf blades, but also on leaf sheaths, stems, glumes, awns and young kernels of susceptible plants (Chen et al., 2014). After seedling stage, uredinia tend to form in stripes, but whole leaves can be covered by uredinia. When leaves are covered by uredinia, photosynthesis is seriously reduced and the continual production of urediniospores sucks water and nutrients from host plants, reducing plant growth, the numbers of tillers and grains per spike and test weight. The disease can cause up to 100% loss of grain yield in fields planted with highly susceptible cultivars under extremely stripe rust favorable weather conditions (Chen, 2005). As *Pst* urediniospores are capable of long-distance dispersal by wind, stripe rust can cause large-scale epidemics. The fungal pathogen evolves fast through mutation, somatic hybridization and even sexual recombination in some regions of the world (Chen and Kang, 2017), producing new races that may overcome race-specific resistance genes deployed in wheat cultivars. Thus, stripe rust is a continual threat to wheat production in all wheat-growing regions of the world (Stubbs, 1985; Chen, 2005; Wang and Chen, 2015; Cheng et al., 2016). Planting resistant cultivars and timely applying fungicides are two major methods for control of stripe rust. However, the former is more economical, easier for farmers and more friendly for the environment (Chen, 2005).

In China, 34 formally named *Pst* races (CYR1 - CYR34) and several dozens of informally named races, so-called “pathotypes” (e.g., Luo-10, Luo-13, Hybrid, Gui-22, and Su-ll), have been identified since the 1950s (Zhan et al., 2011). On average, a new *Pst* race appears in about 1.6 years, while developing a new wheat cultivar needs eight or more years. Since 1950, major wheat cultivars have been replaced eight times in China, mainly because their stripe rust resistances were overcome by new *Pst* races (Liu et al., 2017). Due to the long-term use of a limited number of major genetic stocks in breeding programs, the recent cultivars have a low level of genetic diversity because of their narrow genetic background. The small number of race-specific resistance genes in the current cultivars quickly puts selection pressure on *Pst* for developing new races. For example, wheat cultivar Fan-6 and its derivative cultivars have been widely used in breeding and production in Sichuan province for 30 years, and the emergence of *Pst* race CYR32 and related “pathotypes” have overcome the resistance in the Fan-6 series, leading to several outbreaks of stripe rust. More than 90% of the cultivars with Fan-6 in their pedigrees became susceptible to stripe rust, resulting in yield losses of 120 million kg wheat grain (Li, 2015). More recently, the increase of race CYR34 in the *Pst* population in China, especially in Sichuan province, has circumvented the *Yr26* resistance in many cultivars (Liu et al., 2017). It is urgent to identify new resistance resources and use them in breeding programs for developing resistant cultivars with diverse resistance for sustainable control of stripe rust.

In recent years, genome-wide association studies (GWAS) have been successfully used to provide insights into genetic architecture for phenotypes and to identify quantitative trait loci (QTL) that are significantly associated with stripe rust (Zegeye et al., 2014; Bulli et al., 2016; Zhou et al., 2017; Yao et al., 2019; Liu et al., 2020). Compared to the traditional QTL mapping using bi-parental populations, GWAS can analyze allelic diversity and recombination events present in diverse population panels and identify and map trait-associated QTL in a relatively effective way. To get accurate association loci of interested traits, like stripe rust resistance, using the GWAS approach, it is important to genotype the population using a high-density and high-coverage marker array, as well as to obtain multiple sets of accurate phenotypic data.

Simple sequence repeat (SSR), diversity array technology (DArT) and single-nucleotide polymorphism (SNP) are the main marker technologies commonly used for genotyping (Boukhatem et al., 2002; Chen, 2005; Lan et al., 2010; Zhou et al., 2017; Yao et al., 2020). Compared to other types of markers, SNP markers have relatively high density, capability for high-throughput and commercialization and flexibility, and relatively low cost as they can be easily arranged into arrays or platforms (Sun et al., 2020). To date, the widely used wheat SNP arrays include the Illumina 9K iSelect array (Cavanagh et al., 2013), Illumina 90K iSelect array (Wang et al., 2014), 15K array (Boeven et al., 2016), Axiom 660K array, 55K array, Axiom HD 820K array (Winfield et al., 2016), Breeders’ 35K Axiom array (Allen et al., 2017) and 50K Triticum Trait Breed array (Rasheed and Xia, 2019). In comparison of the seven widely used wheat SNP arrays (excluding the 50K array) in terms of their SNP number, distribution, density, associated genes, heterozygosity and application, Sun et al. (2020) reported that the 660K SNP array contains the highest percentage (99.05%) of genome-specific SNPs with reliable physical positions. The 660K SNP array has been widely used in GWAS and QTL mapping (Wu et al., 2018; Zhou et al., 2018). Thus, we used this array in the present study.

The objectives of this study were to (1) screen Chinese wheat landraces for resistance to stripe rust, (2) map QTL significantly associated with stripe rust resistance using the GWAS approach and the Wheat 660K SNP array and (3) develop KASP markers that can be used for marker-assistant selection (MAS).

## Materials and Methods

### Plant Materials

The wheat panel used in this study consisted of 271 Chinese landrace accessions obtained from the Chinese Academy of Agricultural Sciences. The accessions were originally from 10 wheat production zones of China, as shown in Supplementary Figure 1. The information on name, identification and origin of province and wheat production zones for the landraces, as well as their subpopulations and stripe rust response data obtained in this study, is provided in Supplementary Table 1. Two susceptible lines, Avocet S and SY95-71, from Triticeae Research Institute, Sichuan Agricultural University, were included as susceptible checks in both greenhouse and field tests and also as stripe rust spreaders in the field experiments.

### Field Evaluation of Stripe Rust Resistance at the Adult-Plant Stage

To evaluate the stripe rust response of the wheat landrace panel at the adult-plant stage, field experiments were conducted under artificial inoculation in the 2015–2016 (16CZ), 2016–2017 (17CZ), and 2017–2018 (18CZ) growing seasons in Chongzhou (CZ, 30°32′N, 103°39′E) and in the 2015–2016 (16MY) growing season in Mianyang (MY, 31°48′N, 104°73′E), Sichuan province. All 271 accessions were planted in a randomized block design with three replications at each environment. About 20 seeds were sown in rows of 2.0 m long and 0.3 m apart. Avocet S and SY95-71 were planted every 20 rows as susceptible checks and surrounding the nursery for increasing stripe rust pressure. The mixture of eight *Pst* isolates representing races CYR34, CYR33, CYR32, CYR31, G22-14, Sull-4, Sull-5, and Sull-7 each with an equal quantity of urediniospores was used for inoculating the fields when the plants grew to the fourth leaf stage (Zadoks growth stage 23) (Zadoks et al., 1974). The avirulence/virulence formulae of the isolates are provided in Supplementary Table 2. Disease severity (DS) were recorded three times starting at the boot stage (Zadoks 45) with 7-day intervals as described in our previous study (Yao et al., 2020). Stripe rust infection type (IT) was estimated using the 0–9 scale (Line and Qayoum, 1992). DS was assessed as the percentage of infected leaf, and the final DS at the milk stage (Zadoks 11) was used for various analyses. The area under the disease progress curve (AUDPC) value was calculated for each accession using the three sets of DS data according to the formula: AUDPC = Σ*_*i*_*[(*x*_*i*_ + *x*_*i*+1_)/2]*t*_*i*_, where *x*_*i*_ is the severity value on date *i* and *t*_*i*_ the time in days between dates *i* and *i* + 1 (Lin and Chen, 2007). The IT data of the greenhouse seedling tests and the final IT and DS data together with the AUDPC data calculated from the three sets of DS data of adult-plant stages in the field tests for the 271 Chinese wheat landraces were provided in Supplementary Table 1.

### Greenhouse Evaluation of Stripe Rust Response at the Seedling Stage

The evaluation of the seedling response to stripe rust was carried out in the Gansu Academy of Agriculture Sciences. Two *Pst* races, CYR32 and CYR34, were used in the seedling tests. For each accession, 10–15 seeds were planted in plastic pots of 10 cm in diameter and 10 cm in height and grown in a rust-free growth chamber. After 10–14 days, plants were inoculated with fresh urediniospores mixed with 2% Tween 20 (Sigma-Aldrich, St. Louis, MO, United States) water solution and put in a dew chamber in darkness for 24 h and then transferred to a growth chamber at 14 ± 3°C with 10–14 h of light (660 μmol/m^2^/s) daily. After 18–22 days when *Pst* was fully sporulating on susceptible checks, IT was recorded using the same method as described for the field tests. The resistant accessions with IT 0–3 were re-tested with the same isolate to validate the responses.

### Phenotypic Data Analysis

To display the distribution of stripe rust responses (DS, IT, and AUDPC), violin plots were drawn using the ggplot2 package in the R program V3.6.2 (Wickham et al., 2016). The maximum (Max), minimum (Min), mean, standard deviation (Stdev) and coefficient of variation (CV) values were calculated for each environment. The best linear unbiased estimator (BLUE) value for each trait was calculated using the data across all environments when genotype was considered as a fixed effect in the model using QTL IciMapping (Meng et al., 2015). Pearson correlation coefficients for DS, IT and AUDPC between and across environments were calculated and graphed using the corrplot package in the R program (Wei et al., 2017). The broad-sense heritability (*H*^2^) values of stripe rust responses were estimated for all environments using PROC MIXED COVTEST in SAS V8.0 (SAS Institute Inc., Cary, NC, United States) and formula: *H*^2^ = σ^2^_*G*_/[σ^2^_*G*_ + σ^2^_*E*×*G*_/*n* + σ^2^_*e*_/*rn*], where σ^2^_*G*_ is the variance of genotypes, σ^2^_*G*×*E*_ the variance of the interaction between genotype and environment, σ^2^_*e*_ the variance of residuals, *n* the number of environments and *r* the number of replicates per environment. Genotype, environment and the genotype × environment interaction were treated as random factors (Piepho and Möhring, 2007).

### DNA Extraction and Genotyping

Genomic DNA of the 271 accessions were extracted from seedlings using a modified cetyltrimethylammonium bromide method as described in our previous study (Yao et al., 2019). Genotypic characterization used the Axiom R Wheat 660K SNP array (Affymetrix, Santa Clara, CA, United States). A total of 630,517 probes from the Wheat 660 SNP array (Winfield et al., 2016) were used for genotyping. Markers with 10% missing value were excluded, and only those with minor allele frequencies (MAF) ≥ 0.05 were used for further analyses (Zhou et al., 2017, 2018).

### Population Structure and Linkage Disequilibrium Analyses

The population structure of the wheat panel was analyzed using the compressed mixed linear model as described in the previous study (Zhou et al., 2018), *K*-values ranging from 1 to 10 with a burn-in of 50,000 iterations and 100,000 Monte Carlo Markov chain (MCMC) replicates for the 271 accessions with the selected SNP markers and the Bayesian clustering algorithm in program STRUCTURE V2.3.4 (Pritchard et al., 2000; Falush et al., 2003; Hubisz et al., 2009). The optimal alignment was calculated from Delta K (Δ*K*) statistics using STRUCTURE HARVESTER^1^ (Earl and VonHoldt, 2012). A neighbor-joining tree (NJ-tree) was constructed using software Tassel V3.0 and MEGA7 and visualized using the iTOL website^2^.

After quality control, one marker of every 100 SNP markers were used for LD analysis. LD was measured as squared allele frequency correlations (*r*^2^) among pairs of SNP markers using software TASSEL 3.0^3^ (Bradbury et al., 2007). The pattern of LD decay was then visualized by plotting pairwise *r*^2^ values against the genetic distance (Mb) across the whole genome. Locally weighted polynomial regression curves were fitted into the scatter plot. The physical distance at which the LD decay curve intersects with the critical *r*^2^ value (the point at which the regression curve turns) was used as a threshold to determine the confidence interval of significant QTL (Bulli et al., 2016; Yao et al., 2019).

### Identification of Stripe Rust Resistance Quantitative Trait Loci Using Genome-Wide Association Study

Genome-wide association studies were conducted between the SNP markers and seedling response (IT) and adult-plant response (DS, IT, and AUDPC) of the 271 Chinese wheat landraces. To reduce false-positive associations, a unified mixed linear model (Q + K, MLM) with the Q matrix as the fixed factor and the K matrix as the random factor was implemented in TASSEL 3.0. The exploratory threshold −log10(*P*) ≥ 4.00 (*P* ≤ 0.0001) was used to identify significant marker-trait associations (MTAs) (Zhu et al., 2019). Only MTAs significant in at least three environments were considered for further analyses. MTAs positioned with LD ≥ 0.3 were considered in the same QTL region. Manhattan plots were drawn using the CMplot package in the R program^4^.

### Comparison of Quantitative Trait Loci With Previously Reported Genes and Quantitative Trait Loci for Resistance to Stripe Rust

The physical positions of the QTL detected in the present study were compared with the previously reported *Yr* genes and QTL for resistance to stripe rust using their markers. Their marker positions were referred to the ‘Chinese Spring’ physical map in IWGSC RefSeq V1.0.

### Development and Evaluation of Kompetitive Allele Specific PCR Markers

To make the stripe rust resistance QTL identified in this study more useful in wheat breeding programs, primers for KASP markers representing the significant SNP markers associated with the stable or novel QTL were designed using the PolyMarker software (Ramirez-Gonzalez et al., 2015) and synthesized by TSINGKE Biology Co., Ltd. (Chengdu, China). The KASP markers were validated by testing with 188 accessions selected from the 271 landraces based on their stripe rust phenotypes and presence/absence of the associated SNP marker favorable alleles. The PCR amplification was conducted in a BIO-RAD CFX96 qPCR system using the procedure described in Long et al. (2021). Data analysis was performed manually using the inbuilt BIO-RAD CFX96 Manager v3.1. To determine the polymorphisms of the KASP markers in contemporary cultivars, 94 wheat cultivars from Sichuan province were tested using the same procedure.

## Results

### Seedling and Adult-Plant Resistance of Stripe Rust in the Wheat Landraces

**FIGURE 1:**
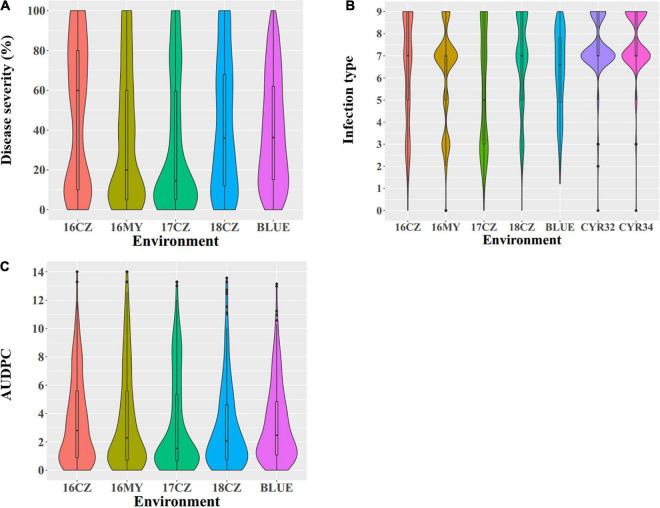
Phenotypic distribution of the 271 Chinese wheat landraces. **(A)** Disease severity (DS, %), **(B)** infection type (IT), and **(C)** area under the disease progress curve (AUDPC). For the environments combined with years and locations, 16 = 2016, 17 = 2017, 18 = 2018; CZ, Chongzhou; MY, Mianyang; and BLUE, best linear unbiased estimator using the data of all environments. CYR32 and CYR34 are races used in the seedling tests.

All phenotypic data are provided in Supplementary Table 1 and summarized in Table 1 while the distributions of the seedling and adult-plant responses are shown in Figure 1. At the seedling stage, the stripe rust response (IT) ranged from 0 to 9 in both tests with races CYR32 and CYR34 in the greenhouse. At the adult-plant stage, the DS values of the 271 Chinese wheat landraces ranged from 0 to 100%, IT 0 to 9 and AUDPC 0 to 14.00, with the mean DS 34.70%, IT 6.08 and AUDPC 2.96. These data indicated significant differences in stripe rust response among the 271 Chinese wheat landraces. The *H*^2^ of final DS (0.90) in the five environments was higher than both IT (0.74) and AUDPC (0.66) (Table 1), indicating the final DS values were relatively stable across environments compared to the IT and AUDPC values.

**TABLE 1:** The stripe rust response summary of the 271 Chinese wheat landraces at the adult plant stage*^a^*.

Trait	Environment	Min	Max	Mean	STDEV	CV	*H* ^2^
Seedling IT	CYR32	0	9	7.42	1.32	0.18	–
	CYR34	0	9	7.67	1.32	0.17	–
AUDPC	16CZ	0	14.00	3.42	3.05	0.89	
	16MY	0	14.00	3.51	3.50	1.00	
	17CZ	0	13.30	3.19	3.40	1.06	0.66
	18CZ	0	13.58	3.01	2.95	0.98	
	BLUE	0	12.50	2.96	2.55	0.86	
DS (%)	16CZ	0	100	46.15	34.27	0.74	
	16MY	0	100	36.50	32.65	0.89	
	17CZ	0	100	31.77	33.00	1.04	0.90
	18CZ	0	100	42.40	32.76	0.77	
	BLUE	0	100	34.70	25.02	0.72	
IT	16CZ	0	9	6.45	2.45	0.38	
	16MY	0	9	6.20	2.12	0.34	
	17CZ	0	9	5.54	2.71	0.49	0.74
	18CZ	0	9	6.80	2.13	0.31	
	19CZ	0	9	6.28	2.19	0.35	
	BLUE	1	9	6.08	1.94	0.32	

*^a^Min, minimum; Max, maximum; STDEV, standard deviation; H^2^, broad-sense heritability; –, not applicable as the test did not have repeats.*

**FIGURE 2:**
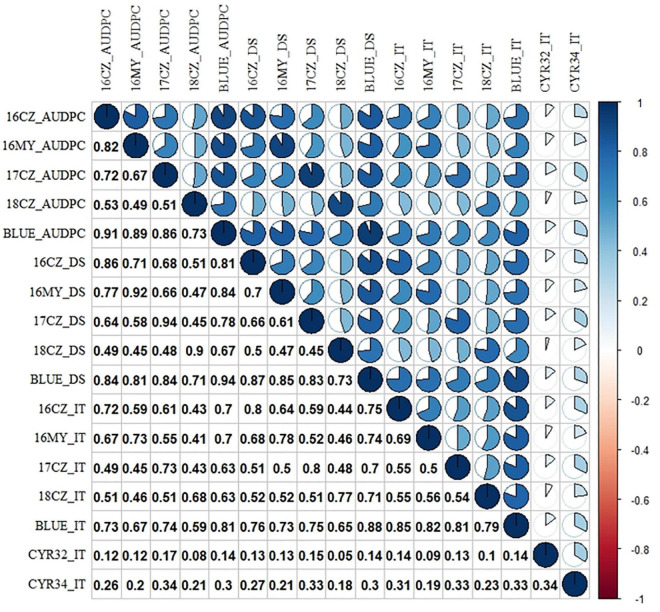
Heatmap of Pearson correlation coefficients among stripe rust response. Positive to negative correlations are displayed in blue to red colors. Color intensity and the scale of the pie chart are proportional to the correlation coefficients. For the environments combined with years and locations, 16 = 2016, 17 = 2017, 18 = 2018; and CZ, Chongzhou; MY, Mianyang; BLUE, best linear unbiased estimator using the data of all environments. IT, infection type; DS, disease severity; and AUDPC, area under the disease progress curve. The IT data were from the seedling tests with races CYR32 and CYR34 of *Puccinia striiformis* f. sp. *tritici*. The *P-*values of the Pearson’s correlation coefficients among the adult-plant stage and between the seeding stage are smaller than 0.001 (*P* < 0.001), while the *P*-values among the seeding stage and adult-plant stage are smaller than 0.05 (*P* < 0.05).

The correlation coefficients among stripe rust responses (DS, IT and AUDPC) for different environments were calculated. The correlation coefficients between seedling and adult-plant stages were low (0.19) as the majority accessions were susceptible in the seedling stage but resistant in the field tests, indicating that the majority landraces have adult-plant resistance. A mean correlation (0.64) between different field environments indicated the relatively consistent stripe rust data across the different growing seasons and locations (Figure 2). Thirteen landraces (Pushanbamai, Liangganbai, Pushanba, Lushanmai, Huayangxiaomai, Zimai, Hongxumai, Qianqianmai, Tiekemai, Huakemai, Mangmai, Laobaimai, and Baichunmai) with stable resistance (IT ≤ 3 and DS ≤ 40%) were identified from the field tests across the five environments (Supplementary Table 1).

### Population Structure and Linkage Disequilibrium of the Landrace Panel

After selection, 178,803 SNP markers with MAF ≥ 5% and a missing rate ≤ 10% were obtained (Supplementary Table 3^5^). The highest number of markers distributed on the B genome (88,293), the lowest number of markers on the D genome (15,229), and the A genome (75,281) in between (Supplementary Table 4). All 178,803 SNP markers were used for the NJ-tree construction and GWAS.

**FIGURE 3:**
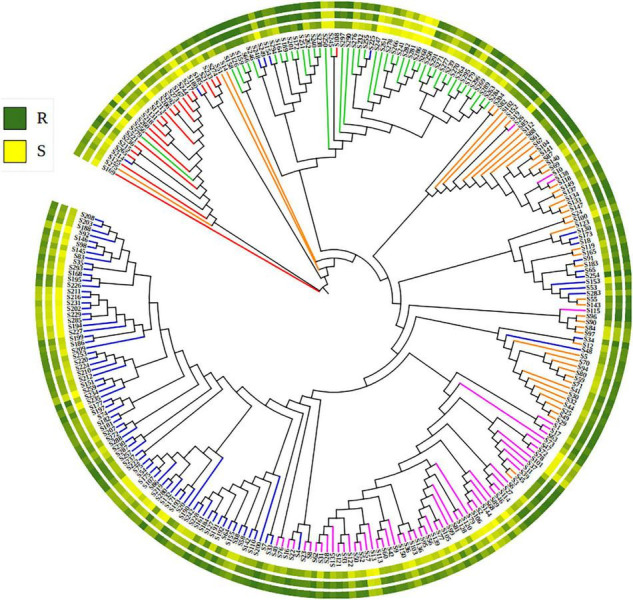
The Neighbor-joining phylogenetic tree showing the phylogenetic relationships of 271 Chinese wheat landraces. Colors of branches corresponding to the five sub-populations: blue (sub-1), purple (sub-2), orange (sub-3), green (sub-4), and red (sub-5). The circle of the colored gradients outside the tree presents the stripe rust response data (BLUE_IT, BLUE_AUDPC, and BLUE_DS). R, resistance and S, susceptible to stripe rust.

The 271 landraces were grouped into five sub-populations: Sub-1 (92), Sub-2 (59), Sub-3 (53), Sub-4 (45), and Sub-5 (23). Sub-1 mainly included landraces from Zone II (55.4%) and Zone I (33.7%). Sub-2 mainly included landraces from Zone III (64.4%), Zone IV (18.6%), and Zone II (10.2%). Sub-3 mainly included landraces from Zone V (44.2%), Zone III (28.8%), and Zone II (17.3%). Sub-4 mainly included landraces from Zone IX (68.9%), Zone VIII (13.3%), and Zone V (11.1%). Sub-5 mainly included landraces from Zone II (26.1%), Zone I (21.7%), Zone V (21.7%), Zone III (13.0%), and Zone IX (13.0%) (Supplementary Table 1). A similar grouping was obtained in the NJ-tree (Figure 3).

**FIGURE 4:**
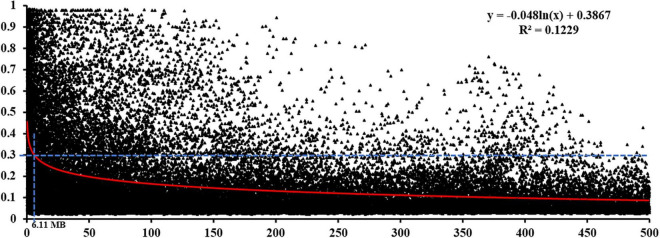
Linkage disequilibrium decay in the Chinese wheat landrace panel. The red curve represents the model fitting the LD decay. The horizontal blue dashed line indicates the standard critical *r*^2^ = value (0.30).

In total, 1,795 markers (one marker from every 100 markers covering all chromosomes) were selected for the LD analysis. The pairwise measure of LD was estimated based on the squared allele frequency correlations (*r*^2^) between every two markers on the same chromosome with their physical distances. At the whole genome level, the LD decay below the critical *r*^2^ = 0.30 was estimated for distances greater than 6.11 Mb (Figure 4), which was used as the confidence intervals to identify significant marker-trait associations. Therefore, the map distance at which LD fell below the LD threshold (*r*^2^ ≥ 0.30) was used to define the confidence intervals of QTL detected in the GWAS analysis, similar to the thresholds reported in previous studies (Bulli et al., 2016; Yao et al., 2019).

### Quantitative Trait Loci for Resistance to Stripe Rust

**FIGURE 5:**
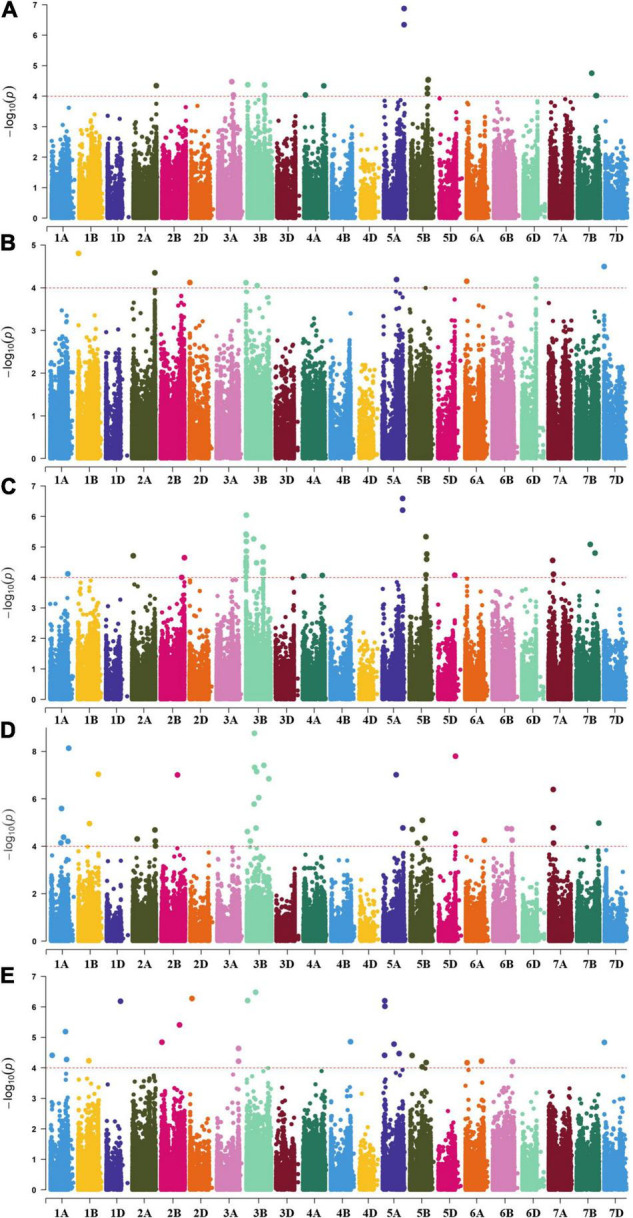
Manhattan plots of –log_10_(*P*) values for markers associated with stripe rust resistance response detected in multiple field experiments. The red dash line had the threshold –log_10_(*P*) value of 4.0 (*P* = 0.0001). Significant associated markers are shown above the lines. **(A)** BLUE_DS, **(B)** BLUE_IT, **(C)** BLUE_AUDPC, **(D)** CYR32_IT, and **(E)** CYR34_IT.

With the threshold −log_10_(*P*) ≥ 4.00, a total of 354 significant MTAs were identified for stripe rust resistance, of which 155 MTAs were detected in more than two environments or located within the LD decay distance (6.11 Mb) (Supplementary Table 5). The 155 MTAs were mapped in 17 genomic regions that were named as 17 QTL: *QYrCL.sicau-1AL*, *QYrCL.sicau-1BL*, *QYrCL.sicau-2AL*, *QYrCL.sicau-2DS*, *QYrCL.sicau-3AL*, *QYrCL.sicau-3BS.1*, *QYrCL.sicau-3BS.2*, *QYrCL.sicau-3BS.3*, *QYrCL.sicau-3B.4*, *QYrCL.sicau-3B.5*, *QYrCL.sicau-3BL.6*, *QYrCL.sicau-5AL.1*, *QYrCL.sicau-5AL.2*, *QYrCL.sicau-5AL.3*, *QYrCL.sicau-5BL*, *QYrCL.sicau-6DL*, and *QYrCL.sicau-7AL*. The 17 QTL were located on 10 chromosomes (1A, 1B, 2A, 2D, 3A, 3B, 5A, 5B, 6D, and 7A) and explained phenotypic variation from 6.06 to 16.46% for DS, IT, or AUDPC. The 17 QTL were detected with three to 36 MTAs. To simplify, only two (at the ends of intervals) or three (at both ends plus one at the middle of the interval) significant markers are presented for each QTL in Table 2. Among the 17 QTL, eight were detected in both seedling and adult-plant stages, and thus considered for all-stage resistance (ASR). The other nine QTL were detected only in the field tests and thus considered for adult-plant resistance (APR). The Manhattan plots in Figure 5 show the significant loci detected in the adult-plant stage BLUE_DS (A), BLUE_IT (B), BLIE_AUDPC (C) and the seedling stage CYR32_IT (E) and CYR34_IT (F).

**TABLE 2:** Stripe rust resistance QTL identified in the 271 Chinese wheat landraces at seedling and adult-plant stages.

QTL	Number of MTAs	Marker	Position (Mb)	Stage	Trait	Marker R^2^ (%)	−log_10_(*P*)	Favorable allele	Effect	References
*QYrCL.sicau-1AL*	4	*AX-109862603*	587.93	Adult	16MY_AUDPC	10.37	5.43	C	−6.10	Bulli et al., 2016
		*AX-109864002*	593.76	Seedling	CYR32_IT	15.23	8.14	G	7.47	
*QYrCL.sicau-1BL*	3	*AX-109429172*	664.08	Seedling	CYR32_IT	13.00	7.03	A	7.46	Bansal et al., 2014; Ye et al., 2019
		*AX-111009273*	665.31	Adult	BLUE_AUDPC	7.52	4.21	G	−5.01	
*QYrCL.sicau-2AL*	13	*AX-108867793*	755.56	Seedling	CYR32_IT	7.68	4.22	A	2.21	Boukhatem et al., 2002
		*AX-109067160*	761.41	Adult	17CZ_DS	9.38	5.08	C	1.11	
		*AX-108886459*	767.51	Adult	17CZ_AUDPC	9.16	4.48	A	−2.95	
*QYrCL.sicau-2DS*	5	*AX-110390887*	16.85	Adult	17CZ_AUDPC	8.56	4.49	C	−4.23	Lu et al., 2009; Naruoka et al., 2015
		*AX-110737036*	24.32	Adult	BLUE_AUDPC	7.32	4.07	G	−2.05	
*QYrCL.sicau-3AL*	3	*AX-109477203*	719.95	Adult	17CZ_AUDPC	9.14	4.82	C	−5.69	New
		*AX-110970789*	724.47	Seedling	CYR34_IT	7.98	4.22	T	2.04	
*QYrCL.sicau-3BS.1*	36	*AX-109977908*	0.34	Adult	BLUE_IT	7.87	4.37	A	−1.89	Khlestkina et al., 2007; Dedryver et al., 2009; Zhao et al., 2012; Yang et al., 2013; Basnet et al., 2014; Case et al., 2014; Lan et al., 2014; Randhawa et al., 2015; Zhou et al., 2015a,b
		*AX-108747357*	0.93	Adult	17CZ_AUDPC	8.05	4.36	C	−1.33	
*QYrCL.sicau-3BS.2*	10	*AX-109818815*	8.80	Adult	16MY_DS	7.67	4.06	A	−40.25	Hao et al., 2011; Lowe et al., 2011;Chen et al., 2012; Lan et al., 2014; Zhou et al., 2015b; Jia et al., 2020;
		*AX-109833897*	11.66	Adult	BLUE_AUDPC	8.62	4.77	G	0.96	
*QYrCL.sicau-3BS.3*	3	*AX-109969055*	40.91	Adult	18CZ_DS	8.49	4.37	C	−22.85	Yao et al., 2019
		*AX-110956592*	43.09	Seedling	CYR34_IT	11.34	6.21	A	6.08	
*QYrCL.sicau-3B.4*	3	*AX-110412110*	256.78	Seedling	CYR32_IT	16.46	8.76	A	5.84	New
		*AX-109532001*	257.82	Adult	18CZ_AUDPC	9.80	5.42	G	−6.48	
*QYrCL.sicau-3B.5*	6	*AX-111760388*	357.24	Adult	18CZ_AUDPC	10.08	5.41	T	2.17	New
		*AX-108920914*	361.45	Adult	18CZ_DS	8.57	4.36	A	25.43	
*QYrCL.sicau-3BL.6*	24	*AX-110532776*	573.40	Adult	BLUE_AUDPC	7.47	4.15	G	1.07	Jighly et al., 2015
		*AX-109826941*	576.05	Seedling	CYR32_IT	13.82	7.42	T	7.46	
		*AX-111667495*	578.59	Adult	16MY_DS	7.53	4.05	A	−56.30	
*QYrCL.sicau-5AL.1*	4	*AX-111070530*	622.55	Adult	18CZ_IT	6.39	4.35	T	0	New
		*AX-108874798*	622.56	Adult	18CZ_IT	6.57	4.29	C	0	
*QYrCL.sicau-5AL.2*	6	*AX-110925235*	663.07	Adult	18CZ_DS	8.43	4.14	T	0.30	Ren et al., 2012
		*AX-109533142*	666.35	Adult	16CZ_AUDPC	11.44	4.95	C	0.33	
		*AX-110673818*	671.19	Adult	BLUE_IT	8.20	4.43	A	0.14	
*QYrCL.sicau-5AL.3*	9	*AX-89474079*	680.86	Adult	16MY_AUDPC	13.59	7.11	A	−6.19	Lan et al., 2010
		*AX-111582891*	680.88	Adult	BLUE_DS	9.10	4.91	T	−5.66	
*QYrCL.sicau-5BL*	10	*AX-110387113*	545.94	Seedling	CYR34_IT	7.54	4.17	T	2.51	Ye et al., 2019
		*AX-109584506*	551.54	Adult	BLUE_AUDPC	6.70	4.50	C	−3.71	
*QYrCL.sicau-6DL*	3	*AX-108822201*	467.03	Adult	16MY_AUDPC	7.52	4.09	G	0.84	Zegeye et al., 2014
		*AX-110991388*	467.04	Adult	17CZ_DS	8.04	4.35	A	0.08	
*QYrCL.sicau-7AL*	13	*AX-110935797*	693.58	Adult	17CZ_DS	7.89	4.34	C	−2.24	New
		*AX-111108248*	693.84	Adult	17CZ_IT	8.44	4.51	C	−2.78	

### Comparison With the Previously Reported *Yr* Genes and Quantitative Trait Loci

Through comparing with the previously reported *Yr* genes and QTL in physical position, five QTL (*QYrCL.sicau-3AL*, *QYrCL.sicau-3B.4*, *QYrCL.sicau-3B.5*, *QYrCL.sicau-5AL.1*, and *QYrCL.sicau-7AL*) were presumably determined to be novel loci for stripe rust resistance (Supplementary Table 5). The remaining twelve were likely the same or tightly linked to previously reported genes or QTL for resistance to stripe rust.

### Distributions of Favorable Alleles of Identified Quantitative Trait Loci in the 271 Chinese Wheat Landraces

**FIGURE 6:**
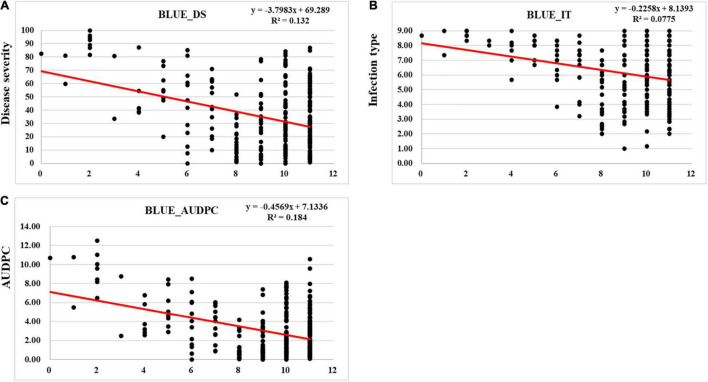
Regression of best linear unbiased estimator (BLUE) using the response to stripe rust in all environments against the number of favorable alleles in 271 Chinese wheat landraces. **(A)** Disease severity (DS), **(B)** infection type (IT), and **(C)** area under the disease progress curve (AUDPC).

We detected 2–14 favorable alleles for stripe rust response (DS, IT, and AUDPC) at the adult-plant stage distributing in the 271 entries (Figure 6 and Supplementary Table 6). With the increase of the favorable allele numbers, the DS, IT, and AUDPC values decreased, indicating that pyramiding more resistance alleles could increase resistance to stripe rust (Figure 6). The 13 stably resistant landraces each had a high number of favorable alleles (7–14) (Supplementary Table 6).

### Kompetitive Allele Specific PCR Markers for Stable and Novel Quantitative Trait Loci

Five SNP markers (*AX-109477203*, *AX-108747357*, *AX-109409794*, *AX-95168494*, and *AX-111108248*) associated with four stable QTL (*QYrCL.sicau-3AL*, *QYrCL.sicau-3BS.1*, *QYrCL.sicau-5AL.1*, and *QYrCL.sicau-7AL*), all of which were presumably new except the first one, were successfully converted to KASP markers (Table 3) and used to test 188 landraces from the GWAS panel and 94 cultivars grown in Sichuan province. The genotyping data are provided in Supplementary Table 7. In the 188 landraces, 90.32–97.33% of the 540 KASP marker data points were consistent to the corresponding SNP data points, indicating that these KASP markers were highly reliable. The frequencies of resistant alleles (60.43 and 76.47%) of *AX-109477203* and *AX-108747357* were higher than those of the susceptible alleles (8.56 and 5.88%, respectively) in the tested landraces. In contrast, *AX-109409794*, *AX-95168494*, and *AX-111108248* had low resistant allele frequencies (5.88, 6.42, and 14.97%, respectively). When the 94 Sichuan cultivars were tested with these five KASP markers, the frequencies of the resistant alleles for QTL on chromosome 3A, 3B, and 5A were very low (1.06–9.57%). These results showed that the resistance QTL were largely absent in the currently grown cultivars and the markers were highly polymorphic, indicating that the KASP markers could be used in MAS for incorporating the QTL into elite wheat cultivars.

**TABLE 3:** Primer squences of KASP markers developed from SNP markers significant associated with stable and novel QTL detected in this study.

KASP	QTL	Primer sequence (5′–3′)
AX-109477203A	*QYrCL.sicau-3AL*	GAAGGTGACCAAGTTCATGCTTGCCTCTCAATGTACATTGCATAG
AX-109477203B	*QYrCL.sicau-3AL*	GAAGGTCGGAGTCAACGGATTTGCCTCTCAATGTACATTGCATAC
AX-109477203C	*QYrCL.sicau-3AL*	CCGTCGGCACTCGTGTATAT
AX-108747357A	*QYrCL.sicau-3BS.1*	GAAGGTGACCAAGTTCATGCTACTTGTGAAACGTTGGGCTTTC
AX-108747357B	*QYrCL.sicau-3BS.1*	GAAGGTCGGAGTCAACGGATTACTTGTGAAACGTTGGGCTTTT
AX-108747357C	*QYrCL.sicau-3BS.1*	GCTTTCCTTTATTGTCCAAGCA
AX-109409794A	*QYrCL.sicau-5AL.1*	GAAGGTGACCAAGTTCATGCTTCATACATTTGAGCCCTGTATTGA
AX-109409794B	*QYrCL.sicau-5AL.1*	GAAGGTCGGAGTCAACGGATTTCATACATTTGAGCCCTGTATTGG
AX-109409794C	*QYrCL.sicau-5AL.1*	CTTCCAATTTCTTCTCTTGAGCC
AX-95168494A	*QYrCL.sicau-5AL.1*	GAAGGTGACCAAGTTCATGCTGGCTGGGTTTCTTTCTCCC
AX-95168494B	*QYrCL.sicau-5AL.1*	GAAGGTCGGAGTCAACGGATTGGCTGGGTTTCTTTCTCCA
AX-95168494C	*QYrCL.sicau-5AL.1*	TCTAGAAGAGCAGAAACCAAGATG
AX-111108248A	*QYrCL.sicau-7AL*	GAAGGTGACCAAGTTCATGCTCTCCTCTATCTGCTCCATCCC
AX-111108248B	*QYrCL.sicau-7AL*	GAAGGTCGGAGTCAACGGATTCTCCTCTATCTGCTCCATCCT
AX-111108248C	*QYrCL.sicau-7AL*	GACCGATGAGACGATGTGCT

## Discussion

Stripe rust occurs throughout the wheat growing regions of the world. In China, the climatic conditions in northwestern Sichuan province and southeastern Gansu province are highly suitable for infection, growth and survival of *Pst*. Because of high stripe rust pressure, stripe rust resistance is a top priority of wheat breeding programs and wheat cultivars developed and grown in these regions are generally resistant to stripe rust at least when released. Due to the long-term selection under the high stripe rust pressure, more wheat landraces from these regions are resistant to the disease than other regions as demonstrated in this study. Among the 13 landraces with stable resistance, 10 originated from Sichuan, Gansu, Shaanxi, Guizhou, and Yunnan, where stripe rust occurs more frequently than in most of the other provinces (Liu et al., 2017).

As the primary gene pool, wheat landraces have high genetic diversity and are rich sources of useful traits including stripe rust resistance. Wheat landraces may have undesirable traits, especially low yield potential and low quality. However, landraces are much easier to use than alien species as they can be easily crossed with elite wheat cultivars. The breeding process can be accelerated by MAS or genomic selection. The 13 landraces with resistance to stripe rust identified in the present study and the markers, especially the KASP markers, can be used to incorporate or pyramid the resistance QTL into new wheat cultivars.

With the high-confidence threshold of −log_10_(*P*) ≥ 4.00, 17 QTL were identified on chromosomes 1A, 1B, 2A, 2D, 3A, 3B, 5A, 5B, 6D, and 7A associated with ASR or APR to stripe rust. These QTL explained a mean of 8.60% of the phenotypic variation. Compared with the previously reported *Yr* genes and QTL, five QTL on chromosomes 3A, 3B, 5A, and 7A were presumably identified as novel loci. The uniqueness or relationships of these QTL with previously reported genes or QTL for stripe rust resistance are discussed below.

*QYrCL.sicau-1AL* was identified as an ASR QTL as it was detected in both the seedling test with race CYR32 (CYR32_IT) and field tests at the adult-plant stage (16CZ/16MY/BLUE_AUDPC). This QTL was mapped between 587.93 and 593.76 Mb on the long arm of chromosome 1A. Bulli et al. (2016) reported a QTL (*QYr.wsu-1A.2*) associated with SNP marker *IWA3215* at the 593.30 Mb position of chromosome 1A, overlapping with the confidence intervals of *QYrCL.sicau-1AL*. Therefore, these two QTL are likely the same. *QYrCL.sicau-1BL* was also identified as an ASR QTL, mapped between 664.08 and 665.31 Mb on chromosome 1B, overlapping with *Qyrsicau-1BL.1* (670.37–670.59 Mb) and *QYr.sun-1B* with marker wPt-1770 at the 671.74 Mb position. As *Qyrsicau-1BL.1* and *QYr.sun-1B* were considered to be *Yr29* for APR (Bansal et al., 2014; Ye et al., 2019), whereas *QYrCL.sicau-1BL* conferred ASR in the present study, the latter should be different from *Yr29*. As many genes conferring ASR to stripe rust have been mapped to chromosome 1B (Wang and Chen, 2017), the relationships to previously reported genes/QTL on 1BL need further studies.

*QYrCL.sicau-2AL* was identified as an ASR QTL and mapped between 755.56 and 767.51 Mb on chromosome 2A, overlapping with *QYR2* close to the SSR *Xgwm356* marker locus (753.5 Mb) (Boukhatem et al., 2002). *QYrCL.sicau-2DS* was associated with 17CZ/BLUE_AUDPC and 16MY/18CZ_IT and mapped at 16.85-24.32 Mb on the short arm of chromosome 2D in the present study. *QYr.caas-2DS* was reported in the SSR marker interval *Xcfd51-Xgwm261* on chromosome 2DS (Lu et al., 2009) and *QYr.wpg-2D.1* identified with SNP marker *IWA1939* (Naruoka et al., 2015), both on chromosome 2D. Based on the map locations using the reference sequence of Chinese Spring (IWGSC RefSeq v1.0), *QYrCL.sicau-2DS* is likely the same as *QYr.caas-2DS* (12.40–19.62 Mb) and *QYr.wpg-2D.1* (20.77 Mb).

*QYrCL.sicau-3AL* was identified as an ASR QTL associated with 17CZ_DS/AUDPC and CYR34_IT and mapped to 719.9–724.5 Mb on chromosome 3AL. Few QTL have been reported on the long arm of chromosome 3A, and they are far away from *QYrCL.sicau-3AL. QYrCL.sicau-3AL* is likely a new locus for resistance to stripe rust. Considering the LD decay distance of 6.11 Mb, six QTL were identified on chromosome 3B, namely *QYrCL.sicau-3BS.1*, *QYrCL.sicau-3BS.2*, *QYrCL.sicau-3BS.3*, *QYrCL.sicau-3B.4*, *QYrCL.sicau-3B.5*, and *QYrCL.sicau-3BL.6*. These six QTL were mapped at the 0.34–0.93, 8.80–11.66, 40.91–43.09, 256.78–257.82, 357.24–361.45, and 573.40–578.59 Mb intervals of chromosome 3B, respectively. Previous studies reported several *Yr* genes and several QTL for resistance to stripe rust on chromosome 3B (Wang and Chen, 2017). SSR marker *Xgwm389* positioned at 0.81 Mb on the distal of chromosome 3B was reported to be linked to *QYrAlt.syau-3BS*, *QYr-3B* and *Yr57* on the short arm of chromosome 3BS (Zhao et al., 2012; Randhawa et al., 2015). *XIWA195* (2.89 Mb on 3BS) was reported to be associated to *QYrbr.wpg-3BS.1* (Case et al., 2014). *Xgwm533* (6.67 Mb on 3BS) is linked to *QYr.cim-3BS*, *QYr.nafu-3BS*, *QYr.inra-3BS*, *QYr.tam-3B*, *QYr.nafu-3BS*, *QYr.cim-3BS.2* and *Yrns-B1* (Khlestkina et al., 2007; Dedryver et al., 2009; Yang et al., 2013; Basnet et al., 2014; Lan et al., 2014; Zhou et al., 2015a,b). *Xbarc133* (7.61 Mb on 3BS) is linked to *QYr.nafu-3BS*, *QYr.cim-3BS.2*, *QYr.ucw-3BS*, and *QYr.uga-3BS.1* (Hao et al., 2011; Lowe et al., 2011; Lan et al., 2014; Zhou et al., 2015b). *IWB12253* (9.1 Mb on 3BS) was reported as a significantly associated marker for *QYr.hbaas-3BS* (Jia et al., 2020), and *XwPt-3921* (13.97 Mb on 3BS) for *QYrrb.ui-3B.1* (Chen et al., 2012). Based on the marker positions, these QTL are all close to *QYrCL.sicau-3BS.1* and *QYrCL.sicau-3BS.2*, making it hard to distinguish among them. Further studies are needed to determine their relationships. *QYrCL.sicau-3BS.3* appeared close to *QYrcl.sicau-3B.5* at position 35.52 Mb on the chromosome 3BS (Yao et al., 2020). *QYrCL.sicau-3B.4* for ASR and *QYrCL.sicau-3B.5* for APR were mapped far away from the previously reported *Yr* genes and QTL on chromosome 3B, and they are likely new loci for resistance to stripe rust. *QYrCL.sicau-3BL.6* was identified as an ASR QTL but overlapped with *QRYr3B.2* for APR (Jighly et al., 2015), and their relationship needs a further study.

Three QTL (*QYrCL.sicau-5AL.1*, *QYrCL.sicau-5AL.2*, and *QYrCL.sicau-5AL.3*) were mapped on the long arm of chromosome 5A. *QYrCL.sicau-5AL.1* was detected at 622.55–622.56 Mb with four markers (*AX-111070530*, *AX-109409794*, *AX-95168494*, and *AX-108874798*) in the 2017–2018 field test at Chongzhou. *QYrCL.sicau-5AL.2* was associated with 16CZ_AUDPC, 18CZ_AUDPC/DS, and BLUE_IT and was located at 663.07–671.19 Mb. *QYrCL.sicau-5AL.3* was detected with *AX-89474079* (680.86 Mb) and *AX-111582891* (680.88 Mb) in five environments and explained the highest phenotype variation (13.59%) at the adult-plant stage among the QTL identified in the present study. The distance between *QYrCL.sicau-5AL.2* and *QYrCL.sicau-5AL.3* were greater than the LD decay distance of 6.11 Mb, and thus were designed as different loci. Several *Yr* genes and QTL were reported on chromosome 5AL. *QYr.caas-5AL.2* was located between *XwPt-1903* and *XwPt-3334* (Ren et al., 2012). *QYr.caas-5AL* was a stable QTL located between *Xwmc410* and *Xbarc261* on chromosome 5AL (Lan et al., 2010). When comparing the physical positions of the markers of the previously reported QTL and the three QTL on the chromosome 5A identified in the present study, we found that *wPt-1903* (666.69 Mb) and *wPt-3334* (666.70 Mb) were close or within the interval of *QYrCL.sicau-5AL.2* (663.07–671.19 Mb) and *Xwmc410* (678.29 Mb) was close to the interval of *QYrCL.sicau-5AL.3* (680.86–680.88 Mb). These results indicate that *QYrCL.sicau-5AL.2* is likely the same as *QYr.caas-5AL.2* and *QYrCL.sicau-5AL.3* the same as *QYr.caas-5AL*. As *QYrCL.sicau-5AL.1* is far away from the previously reported QTL and *Yr* genes, it is likely a new locus. *QYrCL.sicau-5BL* was detected in multiple environments (CYR34_IT, 17CZ_DS, 16MY_AUDPC, and BLUE_AUDPC/DS), identified as an ASR QTL and mapped to 545.94–551.54 Mb on chromosome 5B. Ye et al. (2019) reported an APR QTL, *Qyrsicau-5BL.1*, at 554.58 Mb on the long arm of chromosome 5B in some Chinese landraces. As this QTL is close to the interval of *QYrCL.sicau-5BL* within the LD decay threshold of 6.1 Mb, these two QTL are very likely the same.

*QYrCL.sicau-6DL* was identified with markers *AX-108822201* (16MY_AUDPC) and *AX-110991388* (17CZ_DS/AUDPC) between 467.03 and 467.04 Mb of chromosome 6DL. Zegeye et al. (2014) reported a QTL associated with marker *wsnp_Ex_c62371_62036044* on chromosome 6D at 462.63 Mb less than 5 Mb away from *QYrCL.sicau-6DL*. Therefore, these QTL are likely the same.

*QYrCL.sicau-7AL* was identified with 13 MTAs in the 2017 field test at the Chongzhou location. After comparing its position with the previously reported QTL on 7AL referring to the “Chinese Spring” physical map (IWGSC Refseq V1.0), we concluded that *QYrCL.sicau-7AL* is a novel QTL for resistance to stripe rust.

As shown in Figure 6, the landraces with low numbers of resistance QTL had high levels of stripe rust (DS, IT, and AUDPC) while the landraces with high numbers of resistance QTL had low levels of stripe rust. This indicates that pyramiding multiple loci is necessary to achieve a high level of resistance (Jia et al., 2020). One of the challenges in breeding for stripe rust resistance is the lack of diverse effective resistance genes. In the present study, we identified 13 Chinese wheat landraces carrying known and unknown QTL for resistance to stripe rust. These landraces can be used in breeding programs for improving stripe rust resistance in modern high-yielding cultivars. As reported in the previous studies, the combination of multiple resistance genes with minor or intermediate effects in a cultivar may provide a higher level of resistance to stripe rust (Basnet et al., 2014; Bulli et al., 2016; Liu et al., 2018, 2019, 2020; Mu et al., 2020). This is also confirmed by the present study. Wheat landraces Pushanbamai (S115), Liangganbai (S112), Pushanba (S96), Lushanmai (S104), Hongxumai (S14), Huayangxiaomai (S67), Zimai (S85), Qianqianmai (S66), Tiekemai (S126), Huakemai (S159), Mangmai (S189), Laobaimai (S201), and Baichunmai (S251) showed stable resistance to stripe rust in all field environments. These landraces were found to have most of the favorable alleles.

As usually at high level and often controlled by single major genes, ASR is easy to use in breeding programs, while APR is relatively difficult to use as it is often controlled by QTL with small effects and provides partial resistance. However, APR is more durable than ASR (Chen, 2005). Combining the ASR and APR QTL detected in the present study should be a good approach for developing wheat cultivars with adequate and durable resistance to minimize the damage caused by current and new races of *Pst*. The stable QTL, such as *QYrCL.sicau-2AL*, *QYrCL.sicau-3BS.1*, *QYrCL.sicau-3BS.2*, *QYrCL.sicau-3BL.6*, *QYrCL.sicau-5BL*, and *QYrCL.sicau-7AL*, identified in the present study can be used in the breeding programs. The markers for these QTL could be used in MSA. To develop easy-to-use markers, we converted the significantly associated SNP markers of *QYrCL.sicau-3AL* (*AX-109477203*), *QYrCL.sicau-3BS.1* (*AX-108747357*), *QYrCL.sicau-5AL* (*AX-109409794* and *AX-95168494*), and *QYrCL.sicau-7AL* (*AX-111108248*) to KASP markers. These KASP markers were found to be highly polymorphic in the modern wheat cultivars, making the markers useful in breeding programs. KASP markers can be developed for the other QTL in further studies. With more flexibility than the original SNP markers, the KASP markers can be more easily used in MAS for incorporating and pyramiding genes into new wheat cultivars with durable resistance to stripe rust.

## Conclusion

In this study, wheat landraces from ten wheat production zones in China were tested to identify stripe rust resistance loci using the GWAS approach. From the 271 landraces tested, 13 with stable resistance were identified in all field experiments inoculated with a mixture of multiple races at the adult-plant stage. The resistant responses of the 13 landraces in the field environments contrast to the generally susceptible reactions in the greenhouse seedling tests with two predominant races indicate APR, which is usually durable. Combing the high throughput 660K SNP array with the stripe rust phenotypes, we identified 17 QTL associated with stripe rust resistance. Five of them are potentially new. Five KASP markers for four of the QTL were developed by converting from their significant SNP markers. The KASP markers were validated by testing a subset of the landrace panel and showed high polymorphisms among modern wheat cultivars. This study provides wheat breeding programs with diverse resistant stocks and user-friendly markers, which should facilitate the transfer of multiple genes for stripe rust resistance into elite breeding lines for developing new cultivars with durable resistance to achieve sustainable control of the devastating disease.

## Data Availability Statement

All datasets generated for this study are included in the article/Supplementary Material. The big SNP genotyping data file is deposited in the Figshare website with the link https://doi.org/10.6084/m9.figshare.16934572. Further inquiries can be directed to the corresponding author.

## Author Contributions

GC designed the study and reviewed and edited the manuscript. FY collected the phenotype data, analyzed the data, and wrote the manuscript. FG, LD, LL, HT, YJ, MD, and HL collected the phenotype data. QJ, JW, PQ, HK, WL, JM, ZP, YW, and YZ reviewed the manuscript. XC provided suggestions for the study and revised the manuscript. All authors contributed to the article and approved the submitted version.

## Conflict of Interest

The authors declare that the research was conducted in the absence of any commercial or financial relationships that could be construed as a potential conflict of interest.

## Publisher’s Note

All claims expressed in this article are solely those of the authors and do not necessarily represent those of their affiliated organizations, or those of the publisher, the editors and the reviewers. Any product that may be evaluated in this article, or claim that may be made by its manufacturer, is not guaranteed or endorsed by the publisher.

## Footnotes

1
http://taylor0.biology.ucla.edu/structureHarvester/

2
https://itol.embl.de/

3
http://www.maizegenetics.net

4
https://github.com/YinLiLin/CMplot

5
https://doi.org/10.6084/m9.figshare.16934572

## Supplementary Material

Supplementary Figure 1 The distribution of the 271 Chinese wheat landraces in the ten wheat Zone in China. Zone I: North China Winter Wheat Zone (38 landraces), Zone II: Huang Huai Facultative Wheat Zone (72), Zone III: Middle and Lower Yangtze Valleys Autumn-Sown Spring wheat Zone (59), Zone IV: Southwestern Autumn-sown Spring wheat Zone (11), Zone V: South China Autumn-sown Spring Wheat Zone (38), Zone VI: Southwestern Autumn-Sown Spring Wheat Zone (1), Zone VII: Northern Spring-sown Spring Wheat Zone (4), Zone VIII: Northwestern Spring Wheat Zone (8), Zone IX: Qinghai-Tibet Spring and Winter Wheat Zone (38), and Zone X: Xinjiang Winter and Spring Wheat Zone (2).

Supplementary Table 1 The information of the 271 Chinese wheat landraces (note: 13 accessions showing stable resistance are marked in bold green).

Supplementary Table 2 Virulence and avirulence formulae of the races and pathotypes used in the present study.

Supplementary Table 3 Genotype data of the 271 Chinese wheat landrace.

Supplementary Table 4 The marker number distribution on the 21 chromosomes and A, B, and D genomes.

Supplementary Table 5 Stripe rust resistance QTL identified in the 271 Chinese wheat landraces in seedling and adult plant stage.

Supplementary Table 6 Distribution of significant associated marker alleles of disease severity (DS), infection type (IT), and the area under the disease progress curve (AUDPC) (*P* < 0.0001) in the Chinese wheat landrace panel.

Supplementary Table 7 KASP marker result of 188 wheat landraces and 94 Sichuan wheat cultivars for the stable and new stripe rust resistance QTL.

## References

[B1] Allen A. M. Winfield M. O. Burridge A. J. Downie R. C. Benbow H. R. Gary L. (2017). Characterization of a wheat breeders’ array suitable for high-throughput SNP genotyping of global accessions of hexaploid bread wheat (*Triticum aestivum*). *Plant Biotechnol. J.* 15 390–401. 10.1111/pbi.12635 27627182 PMC5316916

[B2] Bansal U. K. Kazi A. G. Singh B. Hare R. A. Bariana H. S. (2014). Mapping of durable stripe rust resistance in a durum wheat cultivar Wollaroi. *Mol. Breed.* 33 51–59. 10.1007/s11032-013-9933-x

[B3] Basnet B. R. Singh R. P. Ibrahim A. M. H. Herrera-Foessel S. A. Huerta-Espino J. Lan C. (2014). Characterization of *Yr54* and other genes associated with adult plant resistance to yellow rust and leaf rust in common wheat Quaiu 3. *Mol. Breed.* 33 385–399. 10.1007/s11032-013-9957-2

[B4] Boeven P. H. Longin C. F. H. Leiser W. L. Kollers S. Ebmeyer E. Würschum T. (2016). Genetic architecture of male floral traits required for hybrid wheat breeding. *Theor. Appl. Genet.* 129 2343–2357. 10.1007/s00122-016-2771-6 27553082

[B5] Boukhatem N. Baret P. V. Mingeot D. Jacquemin J. M. (2002). Quantitative trait loci for resistance against yellow rust in two wheat-derived recombinant inbred line populations. *Theor. Appl. Genet.* 104 111–118. 10.1007/s001220200013 12579435

[B6] Bradbury P. J. Zhang Z. Kroon D. E. Casstevens T. M. Ramdoss Y. Buckler E. S. (2007). TASSEL: Software for association mapping of complex traits in diverse samples. *Bioinformatics* 23 2633–2635. 10.1093/bioinformatics/btm308 17586829

[B7] Bulli P. Zhang J. Chao S. Chen X. Pumphrey M. (2016). Genetic architecture of resistance to stripe rust in a global winter wheat germplasm collection. *G3* 6 2237–2253. 10.1534/g3.116.028407 27226168 PMC4978880

[B8] Case A. J. Naruoka Y. Chen X. Garland-Campbell K. A. Zemetra R. S. Carter A. H. (2014). Mapping stripe rust resistance in a BrundageXCoda winter wheat recombinant inbred line population. *PLoS One* 9:e91758. 10.1371/journal.pone.0091758 24642574 PMC3958369

[B9] Cavanagh C. R. Chao S. Wang S. Emma B. Stephen S. Kiani S. (2013). Genome-wide comparative diversity uncovers multiple targets of selection for improvement in hexaploid wheat landraces and cultivars. *Proc. Natl. Acad. Sci. USA* 110 8057–8062. 10.1073/pnas.1217133110 23630259 PMC3657823

[B10] Chen J. Chu C. Souza E. J. Guttieri M. J. Chen X. Xu S. (2012). Genome-wide identification of QTL conferring high-temperature adult-plant (HTAP) resistance to stripe rust (*Puccinia striiformis* f. sp. *tritici*) in wheat. *Mol. Breed.* 29 791–800. 10.1007/s11032-011-9590-x

[B11] Chen W. Wellings C. Chen X. Kang Z. Liu T. (2014). Wheat stripe (yellow) rust caused by *Puccinia striiformis* f. sp. *tritici*. *Mol. Plant Pathol.* 15 433–446. 10.1111/mpp.12116 24373199 PMC6638732

[B12] Chen X. M. (2005). Epidemiology and control of stripe rust [*Puccinia striiformis* f. sp. *tritici*] on wheat. *Can. J. Plant Pathol.* 27 314–337. 10.1080/07060660509507230

[B13] Chen X. Kang Z. (2017). *Stripe rust.* Berlin: Springer. 10.1007/978-94-024-1111-9

[B14] Cheng P. Chen X. M. See D. R. (2016). Grass hosts harbor more diverse isolates of *Puccinia striiformis* than cereal crops. *Phytopathology* 106 362–371. 10.1094/PHYTO-07-15-0155-R 26667189

[B15] Dedryver F. Paillard S. Mallard S. Robert O. Trottet M. Negre S. (2009). Characterization of genetic components involved in durable resistance to stripe rust in the bread wheat “Renan.”. *Phytopathology* 99 968–973. 10.1094/PHYTO-99-8-0968 19594316

[B16] Earl A. D. VonHoldt B. M. (2012). STRUCTURE HARVESTER: a website and program for visualizing STRUCTURE output and implementing the Evanno method. *Conserv. Genet. Resour.* 4 359–361.

[B17] Falush D. Stephens M. Pritchard J. K. (2003). Inference of population structure using multilocus genotype data: Linked loci and correlated allele frequencies. *Genetics* 164 1567–1587. 10.1093/genetics/164.4.1567 12930761 PMC1462648

[B18] Hao Y. Chen Z. Wang Y. Bland D. Buck J. Brown-Guedira G. (2011). Characterization of a major QTL for adult plant resistance to stripe rust in US soft red winter wheat. *Theor. Appl. Genet.* 123 1401–1411. 10.1007/s00122-011-1675-8 21830107

[B19] Hubisz M. J. Falush D. Stephens M. Pritchard J. K. (2009). Inferring weak population structure with the assistance of sample group information. *Mol. Ecol. Resour.* 9 1322–1332. 10.1111/j.1755-0998.2009.02591.x 21564903 PMC3518025

[B20] Jia M. Yang L. Zhang W. Rosewarne G. Li J. Yang E. (2020). Genome-wide association analysis of stripe rust resistance in modern Chinese wheat. *BMC Plant Biol.* 20:2693. 10.1186/s12870-020-02693-w 33109074 PMC7590722

[B21] Jighly A. Oyiga B. C. Makdis F. Nazari K. Youssef O. Tadesse W. (2015). Genome-wide DArT and SNP scan for QTL associated with resistance to stripe rust (*Puccinia striiformis* f. sp. *tritici*) in elite ICARDA wheat (*Triticum aestivum* L.) germplasm. *Theor. Appl. Genet.* 128 1277–1295. 10.1007/s00122-015-2504-2 25851000

[B22] Khlestkina E. K. Röder M. S. Unger O. Meinel A. Börner A. (2007). More precise map position and origin of a durable non-specific adult plant disease resistance against stripe rust (*Puccinia striiformis*) in wheat. *Euphytica* 153 1–10. 10.1007/s10681-006-9182-8

[B23] Lan C. Liang S. Zhou X. Zhou G. Lu Q. Xia X. (2010). Identification of genomic regions controlling adult-plant stripe rust resistance in Chinese landrace pingyuan 50 through bulked segregant analysis. *Phytopathology* 100 313–318. 10.1094/PHYTO-100-4-0313 20205534

[B24] Lan C. Rosewarne G. M. Singh R. P. Herrera-Foessel S. A. Huerta-Espino J. Basnet B. R. (2014). QTL characterization of resistance to leaf rust and stripe rust in the spring wheat line Francolin#1. *Mol. Breed.* 34 789–803. 10.1007/s11032-014-0075-6

[B25] Li B. (2015). Application of wheat corner stone parents and innovation of germplasm resource in Sichuan Province. *Sci. Technol. Rev.* 33 66–70.

[B26] Lin F. Chen X. M. (2007). Genetics and molecular mapping of genes for race-specific all-stage resistance and non-race-specific high-temperature adult-plant resistance to stripe rust in spring wheat cultivar Alpowa. *Theor. Appl. Genet.* 114 1277–1287. 10.1007/s00122-007-0518-0 17318493

[B27] Line R. F. Qayoum A. (1992). Virulence, aggressiveness, evolution and distribution of races of *Puccinia striiformis* (the cause of stripe of wheat) in North America, 1968-1987. *US Dep. Agric. Tech. Bull.* 1788:44.

[B28] Liu B. Liu T. Zhang Z. Jia Q. Wang B. Gao L. (2017). Discovery and pathogenicity of CYR34, a new race of *Puccinia striiformis* f. sp. *tritici* in China. *Acta Phytopathol. Sin.* 47 387–681.

[B29] Liu L. Wang M. Feng J. See D. R. Chao S. M. Chen X. (2018). Combination of all-stage and high-temperature adult-plant resistance QTL confers high-level, durable resistance to stripe rust in winter wheat cultivar Madsen. *Theor. Appl. Genet.* 131 1835–1849. 10.1007/s00122-018-3116-4 29797034

[B30] Liu L. Wang M. Zhang Z. See D. R. Chen X. (2020). Identification of stripe rust resistance loci in U.S. spring eheat cultivars and breeding lines using genome-wide association mapping and *Yr* gene markers. *Plant Dis.* 104 2181–2192. 10.1094/PDIS-11-19-2402-RE 32511046

[B31] Liu L. Yuan C. Wang M. See D. R. Zemetra R. S. Chen X. (2019). QTL analysis of durable stripe rust resistance in the North American winter wheat cultivar Skiles. *Theor. Appl. Genet.* 132 1677–1691. 10.1007/s00122-019-03307-2 30796480

[B32] Long L. Yao F. Guan F. Cheng Y.-K. Duan L. Zhao X. (2021). A stable QTL on chromosome 5BL combined with *Yr18* conferring high-level adult-plant resistance to stripe rust in Chinese wheat landrace Anyuehong. *Phytopathology* 2021 1–39. 10.1094/phyto-10-20-0465-r 33599530

[B33] Lowe I. Jankuloski L. Chao S. Chen X. See D. Dubcovsky J. (2011). Mapping and validation of QTL which confer partial resistance to broadly virulent post-2000 North American races of stripe rust in hexaploid wheat. *Theor. Appl. Genet.* 123 143–157. 10.1007/s00122-011-1573-0 21455722 PMC4761445

[B34] Lu Y. Lan C. Liang S. Zhou X. Liu D. Zhou G. (2009). QTL mapping for adult-plant resistance to stripe rust in Italian common wheat cultivars Libellula and Strampelli. *Theor. Appl. Genet.* 119 1349–1359. 10.1007/s00122-009-1139-6 19756474

[B35] Meng L. Li H. Zhang L. Wang J. (2015). QTL IciMapping: integrated software for genetic linkage map construction and quantitative trait locus mapping in biparental populations. *Crop J.* 3 269–283. 10.1016/j.cj.2015.01.001

[B36] Mu J. Liu L. Liu Y. Wang M. See D. R. Han D. (2020). Genome-wide association study and gene specific markers identified 51 genes or QTL for resistance to stripe rust in U.S. winter wheat cultivars and breeding lines. *Front. Plant Sci.* 11:998. 10.3389/fpls.2020.00998 32719705 PMC7350909

[B37] Naruoka Y. Garland-Campbell K. A. Carter A. H. (2015). Genome-wide association mapping for stripe rust (*Puccinia striiformis* f. sp. *tritici*) in US Pacific Northwest winter wheat (*Triticum aestivum* L.). *Theor. Appl. Genet.* 128 1083–1101. 10.1007/s00122-015-2492-2 25754424

[B38] Piepho H. P. Möhring J. (2007). Computing heritability and selection response from unbalanced plant breeding trials. *Genetics* 177 1881–1888. 10.1534/genetics.107.074229 18039886 PMC2147938

[B39] Pritchard J. K. Stephens M. Rosenberg N. A. Donnelly P. (2000). Association mapping in structured populations. *Am. J. Hum. Genet.* 67 170–181. 10.1086/302959 10827107 PMC1287075

[B40] Ramirez-Gonzalez R. H. Uauy C. Caccamo M. (2015). PolyMarker: A fast polyploid primer design pipeline. *Bioinformatics* 31 2038–2039. 10.1093/bioinformatics/btv069 25649618 PMC4765872

[B41] Randhawa M. S. Bariana H. S. Mago R. Bansal U. K. (2015). Mapping of a new stripe rust resistance locus *Yr57* on chromosome 3BS of wheat. *Mol. Breed.* 35 1–8. 10.1007/s11032-015-0270-0

[B42] Rasheed A. Xia X. (2019). From markers to genome-based breeding in wheat. *Theor. Appl. Genet.* 132 767–784. 10.1007/s00122-019-03286-4 30673804

[B43] Ren Y. He Z. Li J. Lillemo M. Wu L. Bai B. (2012). QTL mapping of adult-plant resistance to stripe rust in a population derived from common wheat cultivars Naxos and Shanghai 3/Catbird. *Theor. Appl. Genet.* 125 1211–1221. 10.1007/s00122-012-1907-6 22798057

[B44] Stubbs R. W. (1985). *Stripe Rust in Diseases, Distribution, Epidemiology, and Control.* Amsterdam: Elsevier, 61–101. 10.1016/b978-0-12-148402-6.50011-0

[B45] Sun C. Dong Z. Zhao L. Ren Y. Zhang N. Chen F. (2020). The Wheat 660K SNP array demonstrates great potential for marker-assisted selection in polyploid wheat. *Plant Biotechnol. J.* 18 1354–1360. 10.1111/pbi.13361 32065714 PMC7206996

[B46] Wang M. Chen X. (2015). Barberry does not function as an alternate host for *Puccinia striiformis* f. sp. *tritici* in the U. S. Pacific Northwest due to teliospore degradation and barberry phenology. *Plant Dis.* 99 1500–1506. 10.1094/PDIS-12-14-1280-RE 30695954

[B47] Wang M. Chen X. (2017). “Stripe rust resistance,” in *Stripe Rust*, eds Chen X. Kang Z. (Berlin: Springer), 353–558.

[B48] Wang S. Wong D. Forrest K. Allen A. Chao S. Huang B. E. (2014). Characterization of polyploid wheat genomic diversity using a high-density 90 000 single nucleotide polymorphism array. *Plant Biotechnol. J.* 12 787–796. 10.1111/pbi.12183 24646323 PMC4265271

[B49] Wei T. Simko V. Levy M. Xie Y. Jin Y. Zemla J. (2017). Package ‘corrplot’. *Statistician* 56:e24.

[B50] Wickham H. Chang W. Wickham M. H. (2016). *Package ‘ggplot2’. Creat. Elegant Data Vis. Using Gramm. Graph. Version 2.* 1–189.

[B51] Winfield M. O. Allen A. M. Burridge A. J. Barker G. L. A. Benbow H. R. Paul A. (2016). High-density SNP genotyping array for hexaploid wheat and its secondary and tertiary gene pool. *Plant Biotechnol. J.* 796 1195–1206. 10.1111/pbi.12485 26466852 PMC4950041

[B52] Wu J. Wang X. Chen N. Yu R. Yu S. Wang Q. (2018). Association analysis identifies new loci for resistance to Chinese *Yr26* -virulent races of the stripe rust pathogen in a diverse panel of wheat germplasm. *Plant Dis.* 104 1751–1762. 10.1094/pdis-12-19-2663-RE 32293995

[B53] Yang E. N. Rosewarne G. M. Herrera-Foessel S. A. Huerta-Espino J. Tang Z. X. Sun C. F. (2013). QTL analysis of the spring wheat “Chapio” identifies stable stripe rust resistance despite inter-continental genotype × environment interactions. *Theor. Appl. Genet.* 126 1721–1732. 10.1007/s00122-013-2087-8 23558982

[B54] Yao F. Long L. Wang Y. Duan L. Zhao X. Jiang Y. (2020). Population structure and genetic basis of the stripe rust resistance of 140 Chinese wheat landraces revealed by a genome-wide association study. *Plant Sci.* 301:110688. 10.1016/j.plantsci.2020.110688 33218646

[B55] Yao F. Zhang X. Ye X. Li J. Long L. Yu C. (2019). Characterization of molecular diversity and genome-wide association study of stripe rust resistance at the adult plant stage in Northern Chinese wheat landraces. *BMC Genet.* 20:736. 10.1186/s12863-019-0736-x 30914040 PMC6434810

[B56] Ye X. Li J. Cheng Y. Yao F. Long L. Yu C. (2019). Genome-wide association study of resistance to stripe rust (Puccinia striiformis f. sp. tritici) in Sichuan wheat. *BMC Plant Biol.* 19:17644. 10.1186/s12870-019-1764-4 30991940 PMC6469213

[B57] Zadoks J. C. Chang T. T. Konzak C. F. (1974). A decimal code for the growth stages of cereals. *Weed Res*. 14 415–421. 10.1111/j.1365-3180.1974.tb01084.x

[B58] Zegeye H. Rasheed A. Makdis F. Badebo A. Ogbonnaya F. C. (2014). Genome-wide association mapping for seedling and adult plant resistance to stripe rust in synthetic hexaploid wheat. *PLoS One* 9:e105593. 10.1371/journal.pone.0105593 25153126 PMC4143293

[B59] Zhan G. Wang J. Wang X. Huang L. Kang Z. (2011). Evolution and genetic recombination of physiological races of *Puccinia striiformis* f. sp. *tritici* in China. *J. Integr. Agric.* 44 1815–1822.

[B60] Zhao L. Feng J. Zhang C. Xu X. Chen X. Sun Q. (2012). The dissection and SSR mapping of a high-temperature adult-plant stripe rust resistance gene in American spring wheat cultivar Alturas. *Eur. J. Plant Pathol.* 134 281–288. 10.1007/s10658-012-9987-3

[B61] Zhou X. L. Zhang Y. Zeng Q. D. Chen X. M. Han D. J. Huang L. L. (2015b). Identification of QTL for adult plant resistance to stripe rust in Chinese wheat landrace Caoxuan 5. *Euphytica* 204 627–634. 10.1007/s10681-014-1349-0

[B62] Zhou X. Han D. Chen X. Mu J. Xue W. Zeng Q. (2015a). QTL mapping of adult-plant resistance to stripe rust in wheat line P9897. *Euphytica* 205 243–253. 10.1007/s10681-015-1447-7

[B63] Zhou Y. Chen Z. Cheng M. Chen J. Zhu T. Wang R. (2018). Uncovering the dispersion history, adaptive evolution and selection of wheat in China. *Plant Biotechnol. J.* 16 280–291. 10.1111/pbi.12770 28635103 PMC5785339

[B64] Zhou Y. Tang H. Cheng M. Dankwa K. O. Chen Z. Li Z. (2017). Genome-wide association study for pre-harvest sprouting resistance in a large germplasm collection of chinese wheat landraces. *Front. Plant Sci.* 8:401. 10.3389/fpls.2017.00401 28428791 PMC5382224

[B65] Zhu Y. Wang S. Wei W. Xie H. Liu K. Zhang C. (2019). Genome-wide association study of pre-harvest sprouting tolerance using a 90K SNP array in common wheat (*Triticum aestivum* L.). *Theor. Appl. Genet.* 132 2947–2963. 10.1007/s00122-019-03398-x 31324930

