# Supplementary figures and images for: Genome-Wide Association Analysis of Stable Stripe Rust Resistance Loci in a Chinese Wheat Landrace Panel Using the 660K SNP Array
Source: Front Plant Sci. 2021 Dec 22;12:783830. doi: 10.3389/fpls.2021.783830 (PMC8728361; doi:10.3389/fpls.2021.783830)

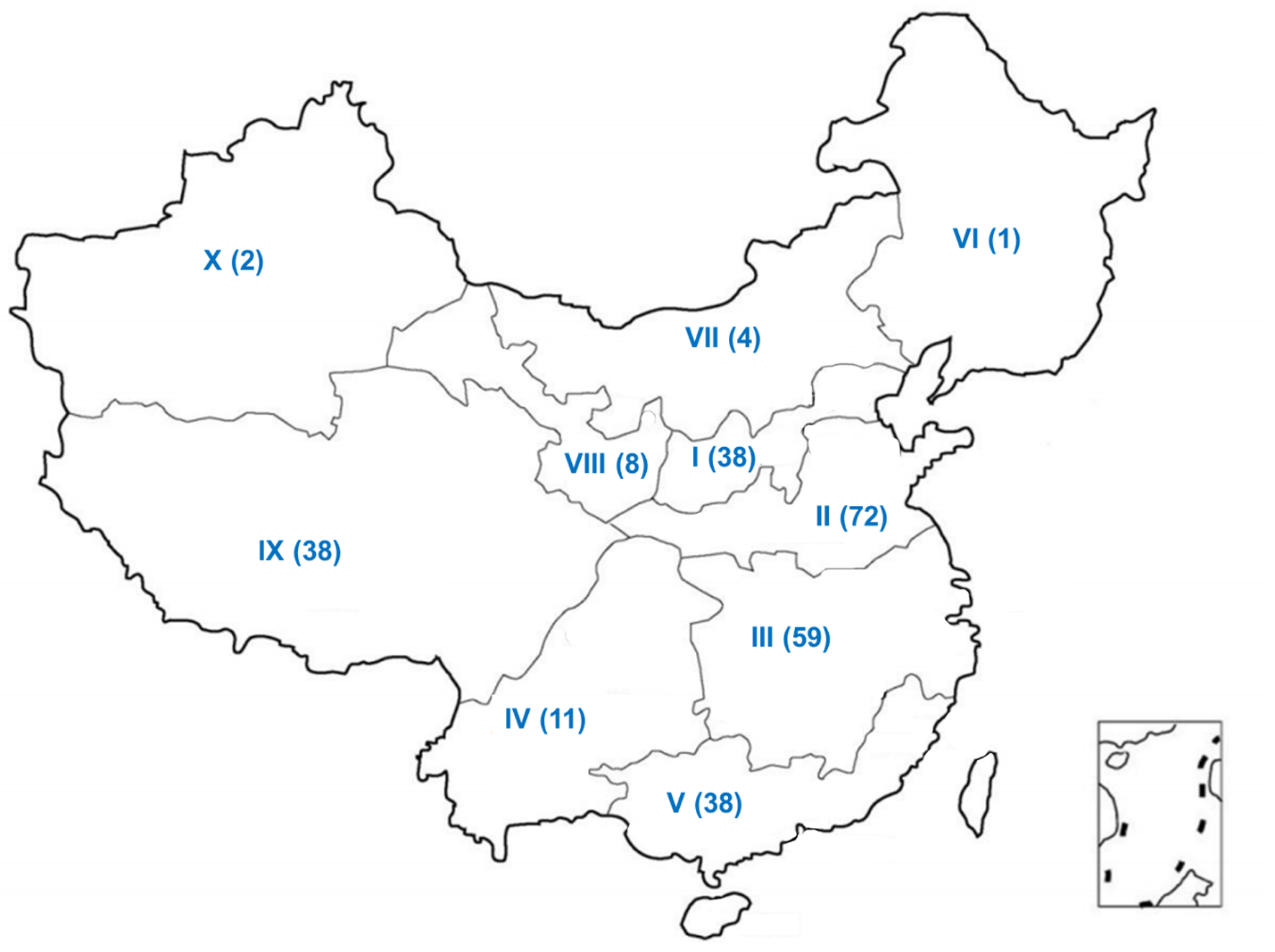

Supplement: Supplementary Figure 1 — The distribution of the 271 Chinese wheat landraces in the ten wheat Zone in China. Zone I: North China Winter Wheat Zone (38 landraces), Zone II: Huang Huai Facultative Wheat Zone (72), Zone III: Middle and Lower Yangtze Valleys Autumn-Sown Spring wheat Zone (59), Zone IV: Southwestern Autumn-sown Spring wheat Zone (11), Zone V: South China Autumn-sown Spring Wheat Zone (38), Zone VI: Southwestern Autumn-Sown Spring Wheat Zone (1), Zone VII: Northern Spring-sown Spring Wheat Zone (4), Zone VIII: Northwestern Spring Wheat Zone (8), Zone IX: Qinghai-Tibet Spring and Winter Wheat Zone (38), and Zone X: Xinjiang Winter and Spring Wheat Zone (2). [file Image_1.TIF]
